# Gender and age differences among current smokers in a general population survey

**DOI:** 10.1186/1471-2458-5-57

**Published:** 2005-06-03

**Authors:** Ulrich John, Monika Hanke, Christian Meyer, Anja Schumann

**Affiliations:** 1 Institute of Epidemiology and Social Medicine, University of Greifswald, Walther-Rathenau-Str. 48, D-17487 Greifswald, Germany

## Abstract

**Background:**

Evidence suggests a higher proportion of current smokers among female than among male ever smokers at the age above 50. However, little is known about the proportion of current smokers among ever smokers in old age groups with consideration of women in comparison to men from general population samples. The goal was to analyze the proportions of current smokers among female and among male ever smokers including those older than 80.

**Methods:**

Cross-sectional survey study with a national probability household sample in Germany. Data of 179,472 participants aged 10 or older were used based on face-to-face in-home interviews or questionnaires. The proportions of current smokers among ever smokers were analyzed dependent on age, age of onset of smoking and cigarettes per day including effect modification by gender.

**Results:**

Proportions of current smokers tended to be larger among female than among male ever smokers aged 40 or above. Women compared to men showed adjusted odds ratios of 1.7 to 6.9 at ages 40 to 90 or older in contrast to men. No such interaction existed for age of onset of smoking or cigarettes per day.

**Conclusion:**

Special emphasis should be given to current smokers among the female general population at the age of 40 or above in public health intervention.

## Background

Quitting smoking is a major public health goal throughout the life span. Benefits of smoking cessation exist even at the age of over 60 years [1,2]. Although there is large and consistent evidence that the proportion of current smokers declines by age including those above 65 [3-7], there are substantial subpopulations that maintain this health detrimental behavior [6]. Even among the oldest olds, individuals maintain smoking [8]. Little is known about associations with gender in this process. Whereas in 1994 the male population in the USA had higher proportions of current smokers than the female population, women and men aged 65 or older did not differ in their proportions of current smokers (female current smokers: 11.1 %, 95 % confidence interval, CI, 9.8 – 12.4; male current smokers: 13.2 %, CI 11.3 – 15.1 %) [3]. One main outcome of public health intervention is the quit rate, expressed as the proportion of former among the ever smokers in a specified period of time. An alternative measure is the proportion of current smokers among ever smokers, especially when exploring factors that may be barriers for quitting.

The proportion of current smokers among male ever smokers aged 65 or older in the USA was 18.5 % (CI 15.0 – 21.0 %) in 1994, which was lower than the equivalent proportion among female ever smokers aged 65 or more (29.3 %; CI 26.1 – 32.5 %) [3]. Female and male smokers did not differ according to cessation rates. However, female smokers compared to male smokers had an odds ratio of 1.9 for relapse in a study about the prediction of smoking cessation and relapse among individuals aged over 50 years [9]. In an Italian general population sample of individuals aged 65 to 84 the proportion of current smokers among female ever smokers was 51.9 % at age 65 – 69 and 36.1 % at age 80 – 84, and among male ever smokers 34.1 % at age 65 – 69 and 20.7 % at age 80 – 84 [6]. In Great Britain, a trend towards lower decrease in the proportion of current smokers among female ever smokers than among male ever smokers was observed based on household survey data using birth cohorts from 1897 to 1951 (age range 25 – 83) [4]. With increasing age, the proportion of current smokers tended to be higher among female than among male ever smokers [4]. Data from Finland, although limited to the age of 64 or younger, revealed a lower rate of individuals who stopped smoking among the female population at age 60 – 64 in comparison to the male population aged 60 – 64 [10].

One reason for the higher proportion of current smokers among older female compared to older male ever smokers might be that the women may be lighter smokers than men and thus are less prone to smoking-attributable disease. Women at a certain age might have smoked less over the lifetime and might have started smoking later in life than men at that age. Female smokers might feel less burden from consequences of smoking compared to men. This assumption is supported by evidence showing a lower tobacco-attributable mortality and fewer years of potential life lost from smoking in women than in men [11]. Accordingly, current smokers among women might fear less threat from disease and death that is attributable to smoking.

Altogether, little is known about the proportion of individuals who maintain smoking in old age among ever smokers with consideration of women compared to men from general population samples, particularly from countries with little activity in smoking prevention. Studies done so far did not include substantial numbers of female and male smokers aged above 65 years, did not focus on proportions of current smokers among ever smokers or did not consider effect modification by gender in a multivariate data analysis. The goal of the present paper was to analyze the proportions of current smokers among female and among male ever smokers across an age range of 10 or older in a general population sample.

## Methods

### Sample

We used the "Mikrozensus", a cluster household sample representative for residents in Germany across the whole age range [12]. Data were collected in April 1999. At that time Germany was a country with very few activities in smoking prevention. Accordingly, there was an underdeveloped anti-smoking climate in the nation [13] and a lower intention to quit smoking compared to countries with more activities in smoking prevention [14]. Every member of each selected household was included into the study as a participant. Participation was mandatory by law for a core part of the assessment, mainly including questions about demographic data, housing and employment. Of the eligible individuals, 97.4 % participated in the core part of the study (N = 724,139). An additional, voluntary part included questions about tobacco smoking in a 45 % random subsample. Available for scientific use were the data of a 70 % random subsample of the 724,139 participants from the German Federal Statistical Office (N = 506,897) [12]. Among this subsample, 232,397 (45.8 %) had participated in the voluntary part of the Mikrozensus. We excluded children aged less than 10 years (n = 22,156). Among the remaining individuals, there were 30,769 with missing data for smoking status. They were excluded from the data analysis as well. Subjects with and without information about the smoking status did not differ in gender, age, school education and having been ill during the last four weeks prior to the interview, when effect sizes are considered. Among those without information about the smoking status, 52.7 % were women, and among those with information about smoking status 52.1 % were women (Likelihood chi^2 ^3.0; not significant; Cohen's w .004 [15]). Among women without information about smoking the mean age was 45.5 (Standard deviation, Std, 21.9), and among women with information about smoking status it was 46.7 (Std 20.7; t-test; p < .001; d 0.06 [15]). Among women without information about the smoking status, there were 17.4 % aged 70 or older, and among women with information about the smoking status this figure was 16.6 (Likelihood chi^2 ^632.3; p < .001; Cohen's w .08 [15]). Among men without information about smoking the mean age was 41.0 (Std 18.9), and among men with information about the smoking status it was 43.6 (Std 19.1; t-test, p < .001; d 0.13 [15]). Among men without information according to smoking status, there were 8.1 % at the age of 70 or older, among men with information about smoking status 9.9 % (Likelihood chi^2 ^420.0; p < .001; Cohen's w .06, [15]). For school education Cohen's w was .06 for women and .05 for men, for having had a disease during the last four weeks prior to the interview Cohen's w was .02 for women and .03 for men indicating no effect. The final sample consisted of 179,472 residents, among them 78,959 ever smokers (44.0 %). The data of these female and male ever smokers were used in our analysis.

The final sample is representative for the general population of Germany aged 10 or older with respect to gender and age [16]: In the 10 year age groups, the maximum deviance of the proportion of women in the final sample from the proportion of women in the same 10 year age groups of the general population was 2.4 percentage points (mean deviance: 1.0 percentage points). The maximum deviance in the distribution of gender over the 10 year age groups was 1.3 percentage points (mean deviance: 0.6 percentage points) among women, among men the maximum deviance was 1.7 percentage points (mean deviance: 0.7 percentage points).

### Assessments

Every person of the household able to understand and to answer the interview questions and present in the household when the interviewer showed up responded in the face-to-face interview. The persons gave information about those household members that were not present. If nobody could be contacted personally the interviewer left a questionnaire to be filled in for all household members. The interview and the questionnaire included the same questions. According to smoking, the individuals were asked: "Are you currently a smoker?" (Yes, regularly/Yes, occasionally/No), if not: "Did you smoke in the past?" (Yes, regularly/Yes, occasionally/No). If the respondent answered "Yes" to one of the two questions s/he was asked: "How old have you been when you started smoking?" (age in years). "What do/did you smoke predominantly?" (cigarettes, cigars, pipe tobacco). "How many cigarettes do you/did you smoke per day?" (less than 5, 5 – 20, 21 – 40, more than 40). The data included the age of the respondent in years, and those aged 95 or older as one group. The individuals were asked "Have you been ill during the last 4 weeks (including today) or have you been injured by accident?" (Yes, ill/Yes, injured by accident/No), if yes: "How long did your illness or your injury last? (7 categories that were recoded to: 1 year or less, more than 1 year). School education was assessed by the respondent's school graduation (not including university degrees; degrees 1–5 for lowest to highest education). Income per individual was assessed by 18 categories.

### Statistical analysis

For the bivariate data analysis, means with standard deviations, proportions and Likelihood chi^2 ^tests were used. For proportions, the effect size estimate Cohen's w was included, and values .10 or higher were interpreted as indicating an effect [15]. For means, the effect size measure d was used with values 0.20 to 0.49 interpreted as indicating a small and values 0.50 to 0.79 indicating a medium effect [15]. For the multivariate data analysis, logistic regression analysis was performed. SPSS 13.0 was used for all analyses.

## Results

### Proportion of current smokers

According to the bivariate data analysis, among the 179,472 residents there were 78,959 ever smokers (44.0 %). Among the 93,588 women, there were 33.4 %, among the 85,884 men 55.6 % ever smokers, more among men than among women (Chi^2 ^9031.6; df 1; p < .001; w .23). The female ever smokers included 64.0 %, the male ever smokers 58.7 % current smokers (Chi^2 ^224.3; df 1; p < .001; w .05). For age groups below 40, the data revealed lower proportions of current smokers among the female ever smokers (Table 1). For age groups 40 or older, there were higher proportions of current smokers among female than among male ever smokers with effects in the age groups between 60 and 89 years. No effect existed among the age groups 10 – 19 and 30 – 59.

**Table 1:** Percent current smokers among female and male ever smokers*

	Women		Men		Chi^2^		P		w
							
	n ever smokers	% current smokers within ever smokers	n ever smokers	% current smokers within ever smokers					
Total	31228	64.0	47731	58.7	224.3		.001		.05
Age									
10 – 19	1396	92.9	1803	95.5	9.4		.01		.06
20 – 29	4770	78.2	5784	85.6	99.2		.001		.10
30 – 39	8084	69.3	9760	74.2	51.4		.001		.05
40 – 49	6988	64.8	9463	64.1	0.8		ns		
50 – 59	4567	56.6	8149	50.7	40.9		.001		.06
60 – 69	2866	48.7	7334	37.2	112.2		.001		.10
70 – 79	2049	34.4	4340	22.3	103.0		.001		.13
80 – 89	468	29.7	1004	19.5	18.2		.001		.11
90 or older	40	30.0	94	20.2	1.5		ns		
Age of onset of smoking									
10 – 15	5543	72.2	9793	64.8	90.5		.001		.08
16	5219	66.0	9258	62.2	20.8		.001		.04
17 – 18	7621	62.4	12796	55.6	90.5		.001		.07
19 – 20	4618	60.6	6506	52.8	67.6		.001		.08
21 – 25	3023	57.0	3806	54.7	3.6		ns		
26 – 70	2717	59.2	1911	54.3	11.2		.001		.05
Cigarettes per day									
less than 5	6894	52.0	5431	52.6	0.4		ns		
5 – 20	20083	68.4	29398	61.4	256.0		.001		.07
21 – 40	2625	72.7	7567	62.6	89.0		.001		.09
41 or more	272	50.0	1200	34.8	21.2		.001		.12
Disease (last 4 weeks)									
not diseased	27042	65.4	40755	60.5	163.4		.001		.05
diseased for 1 year or less	2074	58.9	3163	55.6	5.7		.05		.03
diseased for more than 1 year	1465	47.4	2748	37.6	37.3		.001		.09

* by age, age of onset of smoking, cigarettes per day and disease status.

Chi^2 ^Likelihood chi^2^ test result.

P Significance level: .001 ≤ .001, .01 ≤ .01, .05 ≤ .05, ns not significant.

w Effect size measure Cohen's w [15].

The mean age at onset of smoking among female ever smokers aged 40 to 59 was higher than among male ever smokers aged 40 to 59 with a small and among those aged 60 or older with a medium effect (Table 2). At the age of 50 or older women disclosed a higher proportion of subjects who smoked 5 – 20 cpd than men with an effect.

**Table 2:** Mean age at onset of smoking and cigarettes per day by age*

	Women		Men			T Chi^2^	P	Effect size
				
	n ever smokers	mean % current smokers within ever smokers	n ever smokers	mean % current smokers within ever smokers	mean difference (standard error)			
Total								
Age								
10 – 19								
mean age at onset (SD) cigarettes per day	1303	15.2 (1.6)	1663	15.4 (1.6)	0.2 (0.1)	-2.7	.01	0.10
4 or less %	430	89.1	496	91.1		1.1	ns	
5 – 20 %	854	95.4	1174	97.8		8.7	.01	.07
21 or more %	55	87.3	71	97.2		4.7	.05	.19
20 – 29								
mean age at onset (SD) cigarettes per day	4419	16.7 (2.6)	5367	16.8 (2.5)	0.1 (0.1)	-1.7	ns	0.04
4 or less %	1104	69.3	796	75.9		10.1	.01	.07
5 – 20 %	3142	81.9	4029	87.7		46.6	.001	.08
21 or more %	356	83.1	741	88.5		5.9	.05	.07
30 – 39								
mean age at onset (SD) cigarettes per day	7493	17.3 (3.4)	8976	17.0 (3.2)	0.2 (0.1)	4.1	.001	0.06
4 or less %	1639	58.3	996	62.9		5.3	.05	.04
5 – 20 %	5365	72.2	6420	75.5		16.3	.001	.04
21 or more %	782	79.8	1831	79.2		0.1	ns	
40 – 49								
mean age at onset (SD) cigarettes per day	6464	18.4 (4.5)	8769	17.5 (3.7)	1.0 (0.1)	13.9	.001	0.23
4 or less %	1248	51.3	836	48.8		1.2	ns	
5 – 20 %	4646	68.0	5674	65.7		6.0	.05	.02
21 or more %	802	72.6	2235	67.8		6.3	.05	.04
50 – 59								
mean age at onset (SD) cigarettes per day	4156	21.1 (6.6)	7454	18.5 (4.8)	2.7 (0.1)	23.0	.001	0.48
4 or less %	911	42.2	729	42.5		0.2	ns	
5 – 20 %	2867	61.8	4736	52.1		69.5	.001	.10
21 or more %	543	61.9	1829	50.8		20.7	.001	.09
60 – 69								
mean age at onset (SD) cigarettes per day	2618	24.3 (9.0)	6825	19.3 (6.1)	5.0 (0.2)	26.2	.001	0.68
4 or less %	702	36.5	803	35.0		0.4	ns	
5 – 20 %	1795	55.0	4310	38.6		138.5	.001	.15
21 or more %	225	48.0	1382	31.6		22.2	.001	.12
70 – 79								
mean age at onset (SD) cigarettes per day	1848	25.4 (10.2)	4060	20.0 (6.6)	5.5 (0.3)	21.1	.001	0.66
4 or less %	662	23.6	601	23.3		0.0	ns	
5 – 20 %	1174	40.5	2515	22.6		123.2	.001	.18
21 or more %	107	33.6	564	15.6		17.1	.001	.17
80 or older								
mean age at onset (SD) cigarettes per day	440	26.5 (11.5)	956	21.4 (7.5)	5.1 (0.6)	8.5	.001	0.55
4 or less %	198	24.2	174	20.1		0.9	ns	
5 – 20 %	240	32.9	540	18.0		20.4	.001	.16
21 or more %	27	51.9	114	9.6		22.0	.001	.44

* Means for current smokers; % current smokers within ever smokers.

T T score for t-test of means.

Chi^2^ Likelihood chi^2^ test result for proportions.

P Significance level: .001 ≤ .001, .01 ≤ .01, .05 ≤ .05, ns not significant.

Effect size Cohen's w for proportions and d for differences between means [15].

SD Standard deviation.

The proportion of current smokers among the ever smokers decreased by age (women: Chi^2^ 2463.6; p < .001; w .28; men: Chi^2^ 9103.7; p < .001; w .42). A lower age of onset of smoking was associated with a lower proportion of current smokers among ever smokers (women: Chi^2^ 297.2; p < .001; w .10; men: Chi^2^ 379.5; p < .001; w .09). Smokers who had been ill during the last four weeks prior to the interview showed a lower proportion of current smokers than those who said that they had not been ill. Those with a disease of 1 year or longer showed a lower proportion of current smokers than those who had been ill for a shorter time (women: Chi^2^ 212.4; p < .001; w .08; men: Chi^2^ 561.1; p < .001; w .11).

### Prediction of current smoking

According to the multivariate data analysis, there was an interaction effect of gender and age for the odds of being a current smoker (Table 3). Women who were 80 to 89 years old had an odds ratio of 4.3 (CI 2.4 – 7.6) to be current smokers compared to men at this age. No interaction was found for the age of onset of smoking, for cigarettes per day or disease with gender.

**Table 3:** Prediction of current smoking; logistic regression analysis

	Current smoker	
	
	Odds ratio	Confidence interval*
Age		
10 – 19	ref.	
20 – 29	0.3	0.25 – 0.40
30 – 39	0.2	0.140 – 0.225
40 – 49	0.1	0.093 – 0.150
50 – 59	0.07	0.055 – 0.088
60 – 69	0.04	0.031 – 0.050
70 – 79	0.02	0.016 – 0.026
80 – 89	0.015	0.011 – 0.020
90 or older	0.013	0.006 – 0.026
Gender		
Male	ref.	
Female	1.2	0.96 – 1.4
Age by gender		
10 – 19, female	ref.	
20 – 29, female	1.3	0.8 – 2.1
30 – 39, female	1.5	0.9 – 2.4
40 – 49, female	1.7	1.1 – 2.8
50 – 59, female	2.2	1.4 – 3.5
60 – 69, female	2.8	1.7 – 4.5
70 – 79, female	3.0	1.9 – 5.0
80 – 89, female	4.3	2.4 – 7.6
90 or older, female	6.9	1.7 – 28.3
Age of onset of smoking		
10 – 15	ref.	
16	1.0	0.92 – 1.04
17 – 18	1.0	0.98 – 1.1
19 – 20	1.2	1.16 – 1.3
21 – 25	1.4	1.3 – 1.5
26 – 70	2.0	1.8 – 2.2
Age of onset of smoking by gender		
10 – 15, female	ref.	
16, female	1.0	0.9 – 1.1
17 – 18, female	1.1	0.96 – 1.2
19 – 20, female	1.2	1.04 – 1.4
21 – 25, female	0.9	0.8 – 1.1
26 – 70, female	1.1	0.9 – 1.3
Cigarettes per day		
less than 5	0.54	0.52 – 0.57
5 – 20	ref.	
21 – 40	1.2	1.1 – 1.3
41 or more	0.4	0.38 – 0.5
Cigarettes per day by gender		
less than 5, female	0.7	0.6 – 0.8
5 – 20, female	ref.	
21 – 40, female	1.1	0.97 – 1.2
41 or more, female	1.1	0.8 – 1.5
Disease (last 4 weeks)		
not diseased	ref.	
diseased, 1 year or less	0.8	0.78 – 0.9
diseased, more than 1 year	0.8	0.7 – 0.9
Disease by gender		
not diseased, female	ref.	
diseased, 1 year or less, female	1.0	0.9 – 1.1
diseased, more than 1 year, female	1.0	0.8 – 1.1

N = 58,984 ever smokers.

Logistic regression analysis, odds ratios adjusted for school education (years) and household income per household member. All variables in the table were entered into the model in addition to school education and household income per household member.

* 95 %.

ref. Reference category.

## Discussion

The study reveals two main findings: First, there is a strong gender-age interaction indicating that surviving female ever smokers have higher odds of maintaining smoking at the age of 40 or above than surviving male ever smokers. This difference tends to increase until the age of 90 or above. Our data confirm former results from different countries that suggest higher proportions of current smokers among female than among male ever smokers [3,4,6]. Second, the higher proportion of current smokers among female than among male ever smokers cannot be explained by either a later age of onset of smoking or less cigarettes per day among female smokers according to the multivariate data analysis.

One reason for the female-male difference in the proportion of current smokers among ever smokers may be a selective death rate attributable to smoking or smoking and alcohol risk drinking: Evidence about tobacco-attributable mortality suggests that more male than female current smokers die from tobacco-attributable disease and from tobacco- and alcohol-attributable disease at the age before 65 [11]. However, we do not have data from longitudinal studies including data collection at death about smoking status. Furthermore, evidence shows higher relative risks of obstructive pulmonary disease and vascular disease associated with smoking for women than for men [17], and data revealed that there is myocardial infarction after a lower lifetime dosage of tobacco smoking among female than among male smokers [18]. Altogether, evidence according to selective death rates due to smoking is ambiguous.

A second reason for the female-male difference in the proportion of current smokers among ever smokers may be an age-period-cohort effect showing increasing rates of smoking women since the 1950ies. No data are available about smoking status by age and gender from comparable surveys carried out before 1989. However, lung cancer death rates were considerably higher among men than among women. Among men aged 70 or older, 11,000 lung cancer death cases attributable to smoking occurred compared to 2,600 among women aged 70 or older according to an overview from the year 2000 [19]. It may be assumed that rates of current smokers increased in Germany as in other Western countries. In addition, it may have contributed to the male-female difference of current smokers that the German Nazi regime especially forced health behavior including being abstinent from smoking [20,21]. Therefore, particularly many of the women who had been teenagers or young adults during the Nazi regime may have remained never smokers. The increase of smoking might have been accompanied by less openness towards quitting among female compared to male smokers. Accordingly, female current smokers may have less fear of health disturbance from smoking. If in the general population, particularly among female current smokers, the belief exists that tobacco-attributable disease mainly affects male smokers, then this might lead to lack of concern about the susceptibility to health hazards from smoking among women. Furthermore, there could be biological factors or nicotine dependence that in female more than in male smokers might act as a barrier against quitting. Other potential confounders may include socioeconomic status, education, living in an urban environment, and tobacco advertising that especially addresses sexual attractiveness of young women. However, the odds ratios found in our analysis were adjusted for school education and income per household member.

Women may have been more likely than men to misclassify themselves as never smokers what might have artificially increased the proportion of current smokers among ever smokers [cf. [22]]. This effect might be stronger the longer the time between the smoking and the interview and the fewer cpd the individual has smoked which is more likely for women than for men. Furthermore, recall bias towards misclassification as a never smoker becomes more likely with older age, given the likelihood of age-dependent limitations of memory. On the other hand, the long-term memory is rather little impaired by age. Also, smoking as a young women must have been somewhat outstanding until the 1950ies what could have been easy to recall.

Our findings confirm former results that show a decrease of the proportion of current smoking by age [3-7]. Our data show that this is true even until the age of 80 or older. No potential influence from the perception of having been ill during the last four weeks on the maintenance of smoking could be found, even not from disease that had lasted for more than a year. There may be insufficient understanding of the relationships between single tobacco-attributable disease and its risk among smokers, or there might be psychological coping strategies active for not admitting smoking-related reasons for the disease.

Different kinds of bias may have been introduced by old people. First, it seems more likely to meet old people in their household than middle adult age people that are working. Second, a considerable part of the elderly lives in institutions that were included in the Mikrozensus. Third, there may be more recall bias due to age-related memory deficits. Fourth, women have a higher life expectancy than men. However, these assumptions of a bias are rather unlikely in light of the fact that our final sample was representative for the general population aged 10 or older in Germany with respect to gender and age.

A strength of the data is that they include considerable numbers of respondents in old age due to data gathering at home. On the other hand, there are several limitations to this study. First, it is cross-sectional only, and the data do not allow any conclusions about causal relationships. Second, the interview questions about smoking did not refer to clear time frames. Third, we could not determine the lifetime number of cigarettes smoked or pack-years among former smokers since the interview did not include questions about the date of stopping smoking and there were only 4 categories for the number of cigarettes smoked per day. Fourth, data were based on self-statements only, no validation of smoking was used. However, evidence shows that the proportion of smokers who deny or minimize smoking in survey studies may be negligible and does not significantly change the results [23]. Fifth, sample selection bias may exist due to a large number of missing data for the smoking status. Sixth, the sample is representative for only one country with very little activity in the prevention of tobacco-attributable death and disease at the time of the data collection.

## Conclusion

Special emphasis should be given to current smokers among the female general population at the age of 40 or above. Female smokers might be confronted with more barriers against quitting than male smokers. Prevention activity that is proactively targeted at the female population and tailored to the needs of women at the age of 40 or above might help to stimulate smoking cessation. Proactive population-based intervention focusing on the health hazards of smoking for these women should be used in public health intervention.

## Competing interests

The author(s) declare that they have no competing interests.

## Authors' contributions

UJ carried out the data analysis and wrote major parts of the first draft of the paper. MH obtained the data, introduced the idea of the data analysis, analyzed parts of the data and contributed parts of the text to the paper. CM and AS assisted with the writing of the manuscript and in the interpretation of the results.

## Pre-publication history

The pre-publication history for this paper can be accessed here:


